# Research Relevance and Readiness Among Aging Care Professionals

**DOI:** 10.1093/geroni/igaf122.3432

**Published:** 2025-12-31

**Authors:** Aryn Lee, Jerad Moxley, Jenna Gladfelter, Allison Nickerson, Elaine Wethington, M Carrington Reid, Patricia Kim

**Affiliations:** Cornell University Medical College, New York, New York, United States; Weill Cornell Medicine, New York, New York, United States; LiveOn NY, New York, New York, United States; LiveOn NY, New York, New York, United States; Cornell University, Ithaca, New York, United States; Weill Cornell Medicine, New York, New York, United States; Geriatrics and Palliative Medicine, New York City, New York, United States

## Abstract

Integration of evidence-based programming is central to aging care policy and practice, yet little is known about aging care professionals’ (ACPs) receptivity to research. This study examined research relevance and ACPs’ readiness to translate findings into practice. In collaboration with LiveOn NY, an advocacy organization supporting community-based aging care agencies throughout New York state, we surveyed individuals working in member agencies in 2023. We collected information on respondents’ agencies, priorities, and assessed perceptions of research relevance and readiness using a 5-point Likert scale (1=strongly disagree, 5=strongly agree). Of 1164 email solicitations, 172 (24.78%) ACPs responded, consisting of executives (15.88%), supervisors (40.5%), social workers (24.71%), and case workers (13.53%), averaging 15.5 years of human/aging service experience (SD = 11.79). A regression model analyzed predictors of research relevance and readiness. ACPs rated research as highly relevant (M = 4.4, SD = 0.5). Relevance positively correlated with ACPs’ Hispanic/Latino ethnicity (β=-0.27, p=.003), desire to learn more about research (β = 0.24, p=.01), and research access (β = 0.22, p=.02). However, ACPs reported less readiness to translate research findings into their practices (M = 2.8, SD = 0.68). Readiness was negatively correlated with agencies serving largely multiracial older adults (β=-0.237, p=.01), and positively correlated with desire to learn more about research (β = 0.20, p=.02), lack of support from team members, and ACPs’ agencies to implement evidence-based practices (β = 0.306, p<.001). Despite strong recognition of research relevance, ACPs reported feeling less ready to interpret and integrate research into practice. Addressing barriers (e.g., access and professional development) is crucial for enhancing evidence-based aging care in the community setting.

